# Chromosome-level genome assembly of Dongxiang wild rice (*Oryza rufipogon*) provides insights into resistance to disease and freezing

**DOI:** 10.3389/fgene.2022.1029879

**Published:** 2022-11-15

**Authors:** Zhiqun Que, Qineng Lu, Chunxiu Shen

**Affiliations:** Jiangxi Key Laboratory of Crop Growth and Development Regulation, College of Life Sciences, Resources and Environment Sciences, Yichun University, Yichun, China

**Keywords:** *Oryza rufipogon*, wild rice, *de novo* genome assembly, stress resistance, structural variations

## Abstract

Dongxiang wild rice (DXWR, *Oryza rufipogon* Griff.) belongs to common wild rice *O. rufipogon*, which is the well-known ancestral progenitor of cultivated rice, possessing important gene resources for rice breeding. However, the distribution of DXWR is decreasing rapidly, and no reference genome has been published to date. In this study, we constructed a chromosome-level reference genome of DXWR by Oxford Nanopore Technology (ONT) and High-through chromosome conformation capture (Hi-C). A total of 58.41 Gb clean data from ONT were *de novo* assembled into 231 contigs with the total length of 413.46 Mb and N50 length of 5.18 Mb. These contigs were clustered and ordered into 12 pseudo-chromosomes covering about 97.39% assembly with Hi-C data, with a scaffold N50 length of 33.47 Mb. Moreover, 54.10% of the genome sequences were identified as repeat sequences. 33,862 (94.21%) genes were functionally annotated from a total of predicted 35,942 protein-coding sequences. Compared with other species of *Oryza* genus, the genes related to disease and cold resistance in DXWR had undergone a large-scale expansion, which may be one of the reasons for the stronger disease resistance and cold resistance of DXWR. Comparative transcriptome analysis also determined a list of differentially expressed genes under normal and cold treatment, which supported DXWR as a cold-tolerant variety. The collinearity between DXWR and cultivated rice was high, but there were still some significant structural variations, including a specific inversion on chromosome 11, which may be related to the differentiation of DXWR. The high-quality chromosome-level reference genome of DXWR assembled in this study will become a valuable resource for rice molecular breeding and genetic research in the future.

## Introduction

Rice is a main crop consumed by half the world’s population (Elert, 2014). As the population explosion intensifies and the climate change challenges, the contradiction between large population and food supply is severe day by day, so improving crop yield is urgently needed. In the world, Asian rice *O. sativa* and African rice *Oryza glabarrima* were the common cultivated rice. During the long-term human selection, desirable agronomic traits including bigger seeds, lodging-resistant, and high yield were bred. However, the continuous selection also resulted in decreasing genetic diversity (Sun et al., 2001b; Meyer and Purugganan, 2013), which may be the inadequacy of facing climate change. Fortunately, there were more than twenty wild rice distributed in the world, which displayed abundant geographical, morphological, physiological and genetic diversity (Vaughan et al., 2008).

With the application of sequencing technologies, genome sequences of many species have been decoded. The cultivated rice *O. sativa* was the first crop to assemble the genome sequence, which supplied a useful foundation for functional study and breeding improvement (Yu et al., 2002). Next, more and more cultivated and wild rice were assembled using short-read sequencing and long-read sequencing (Chen et al., 2013; Wang et al., 2014; Wang et al., 2018; Choi et al., 2020; Kou et al., 2020; Li et al., 2021; Panibe et al., 2021; Qin et al., 2021; Zhang et al., 2022), which was helpful for comparative genomics, evolution analysis and variation identification. Genomic variations, including single nucleotide polymorphisms (SNPs, = 1 bp), insertion-deletion (InDels, ≤ 50 bp), and structural variations (SVs > 50 bp), are an important source of genetic diversity in species (Huang et al., 2012; Hollister, 2014; Sun et al., 2018b). Among them, structural variation, including insertion, deletion, inversion, copy number variation and some more complex variants, is a major driving force for the evolution of species, and plays a crucial biological role in rice phenotypic variation (Sun et al., 2018b; Yang et al., 2019a; Ho et al., 2020; Hollox et al., 2022).

The *Oryza* genus includes cultivated rice and wild rice species, Cultivated rice (two species *O*. *sativa* and *O*. *glaberrima*) is domesticated from wild rice species (22 species, including *O. rufipogon*, etc.), and during the domestication process, the diversity of cultivated rice morphological traits is reduced by 40% compared to wild rice species (Xie et al., 2009a; Wang et al., 2014). In addition, the domestication process of rice leads to the loss of several genes associated with biotic and abiotic stress (Xie et al., 2009a). In recent years, many rice disease resistance and stress tolerance genes have been found in wild rice, including genes associated with cold tolerance, insect resistance and other resistance genes (Sun et al., 2001a; Zhang et al., 2006; Huang et al., 2012; Mao et al., 2015; Zhao et al., 2016). Wild rice resources in China are very abundant and widely distributed in central and southern China. Dongxiang wild rice (hereafter DXWR), a wild rice *O. rufipogon* discovered in 1978 in Dongxiang County, Jiangxi Province of China, is thought to be the northernmost distribution (28°14′N latitude and 116°30′E longitude) of any wild rice species (N 28°14′) (Xie et al., 2009b). DXWR is rich in genetic diversity and is a potential source of many genes associated with high yield, hardiness and drought resistance, disease and insect resistance, and cytoplasmic male sterility. The distribution of DXWR was sharply reduced in recent decades. Therefore, DXWR was classified by the Chinese government as the second class of wild relative to food crop for protection. Despite its importance, no reference genome for DXWR has been published to date.

In this study, a high-quality chromosome-level DXWR genome using Nanopore long-read sequencing technology and Hi-C technology was obtained. Comparative genomics and structural variation revealed the reason of DXWR characters, which showed strong resistance to disease and cold. Our reference genome will lay a solid foundation for the molecular breeding of cultivated rice, and develop improved phenotypic rice with high yield, disease resistance and stress resistance.

## Materials and methods

### Plant materials and high-throughput sequencing

DXWR sequenced in this study (*O. rufipogon* Griff.) was planted in the greenhouse of Jiangxi Key Laboratory of Crop Growth and Development Regulation with normal growth conditions. Young leaves (2 cm × 0.3 cm) were collected and immediately frozen in liquid nitrogen, then stored at −80°C. The total genomic DNA from young leaves was extracted using CTAB method (Doyle, 1987). For Illumina DNA paired end (PE) sequencing, library with insert size of 400 bp was constructed by Illumina TruSeq Nano DNA Library Prep Kit and sequenced on Navoseq 6000 instrument (Illumina, San Diego, United States). For Nanopore sequencing, approximately 10 µg of gDNA was size-selected (10–50 kb) and processed using the Ligation sequencing 1D kit (SQK-LSK109, ONT, United Kingdom) according to the manufacturer’s instructions to construct a Nanopore library, and then the library was sequenced on a PromethION sequencer (ONT, United Kingdom) at the Genome Center of Nextomics (Wuhan, China). For Hi-C experiment, the young leaves were fixed with 1% formaldehyde to induce cross-linking (Sigma), and then were lysed and formed the cohesive ends by restriction endonuclease DPN II (NEB). The digested DNA was blunt-ended by filling nucleotides by Klenow enzyme (NEB) with biotin-14-dATP (Invitrogen), then ligated by T4 DNA ligase (NEB). After incubating overnight to reverse cross-links, the ligated DNA was sheared into 300- to 600-bp fragments. The DNA fragments were blunt-end repaired and A-tailed, followed by purification through biotin–streptavidin-mediated pulldown. Finally, Hi-C library was sequenced on Illumina NovaSeq 6000 platform.

### Estimation of genome size and genome assembly

The Illumina PE reads were filtered and used to estimate the genome size and heterozygosity. K-mers were counted with Jellyfish (Kingsford, 2011), and then analyzed with skew normal distribution model and negative binomial model by FindGSE (Sun et al., 2018a) and GenomeScope (Vurture et al., 2017), respectively.

The Nanopore reads with mean quality score more than seven were retained and corrected by NextDenovo (https://github.com/Nextomics/NextDenovo) with specific parameters (read_cutoff = 1k,seed_cutoff = 42k). The corrected reads were assembled into contigs by smartdenvo (-k 17 -J 4000 -d dmo) (Liu et al., 2020a). To acquire more accurate genome, three rounds of correction were performed to the assembled contigs using Racon (Vaser et al., 2017) with nanopore long reads and another four rounds to the corrected genome were applied using NextPolish (Hu et al., 2020) with Illumina short reads.

The qualified Hi-C reads were aligned to the draft genome obtained from the previous step using bowtie2 (v2.3.2) with end-to-end model (-very-sensitive -L 30) (Langmead and Salzberg, 2012). Only the reads that both ends could be uniquely mapped to the draft genome were used in further analysis. Then LACHESIS 14 (https://github.com/shendurelab/LACHESIS) (Burton et al., 2013) according to the agglomerative hierarchical clustering algorithm was used to cluster contigs. The cross-linked maps were visualized and manually checked using Juicebox. Benchmarking Universal Single-Copy Orthologs (BUSCO v3.0.1) (Seppey et al., 2019) and Core Eukaryotic Genes Mapping Approach (CEGMA) (Parra et al., 2007) was used to evaluate the completeness of the assembled genome. Moreover, Illumina PE reads were mapped to the assembled genome using BWA to assess the accuracy. Then single-nucleotide polymorphisms (SNPs) and Indels were called and filtered using SAMtools and bcftools (Li et al., 2009).

### Repeat and non-coding RNA identification

The repeat sequence can be classified as Tandem repeats (TRs) and transposable elements (TEs). TRs were annotated using GMATA (Wang and Wang, 2016) and Tandem Repeats Finder (TRF) (Benson, 1999). A repeat library for DXWR was constructed with the combination of TE.lib, RepMod.lib, and Repbase (Jurka et al., 2005). TE.lib was generated using MITE-hunter (Han and Wessler, 2010), LTR_finder (Xu and Wang, 2007), LTRharverst, and LTR_retriver (Ou and Jiang, 2018); RepMod.lib, a *de novo* repeat library, was generated using RepeatModeler. Then TEs were identified using RepeatMasker (v4.0.6) based on the combined repeat library (Bedell et al., 2000). Finally results of TRs and TEs were merged together.

Additionally, non-coding RNAs were also identified. snRNA and miRNA were obtained using Infernal based on the Rfam (v11.0) database (Griffiths-Jones et al., 2005). rRNA and tRNA were detected by BLAST and tRNAscan-SE (v1.3.1). Then the rRNA and subunits were predicted by RNAmmer (v1.2) (Lagesen et al., 2007).

### Gene prediction and annotation

Gene models were constructed by three methods, *ab initio* prediction, homology-based prediction and RNA-seq-assisted prediction. For the *ab initio* prediction, Augustus (v3.3.1) was used for the *de novo*-based gene prediction with default parameters (Stanke et al., 2006). Meanwhile, proteins of five species (*Oryza brachyantha*, *O. rufipogon*, *Oryza longistaminata*, *Zea mays*, and *Setaria italica*) were used for homology-based prediction through GeMoMa (v1.5.3) with default parameters (Keilwagen et al., 2019). Then, PASA (v2.0.2) was used for the RNAseq-based method of gene prediction, and the RNAseq data was downloaded from NCBI (http://www.ncbi.nlm.nih.gov/geo/query/acc.cgi?acc=GSE73181, SRA number, SRP063832) (Haas et al., 2003). Finally, the results from the three approaches were integrated using EVidenceModeler (EVM v1.1.1) to get the raw gene set (Haas et al., 2008). To obtain precise gene set, genes including transposable elements were filtered with TransposonPSI software (http://transposonpsi.sourceforge.net).

Functional annotation of predicted genes was obtained using two strategies. Firstly, those predicted protein sequences were aligned to SwissProt protein database using Blastp with the best match parameter. The involved pathways of predicted sequences were extracted from the KEGG Automatic Annotation Server (v2.1). Then the annotation of motifs and domains were performed using InterProScan (v5.32–71.0) to search against opening databases including Pfam, ProDom, PRINTS, PANTHER, SMRT, and PROSITE (Blum et al., 2021).

### Whole genome duplication analysis

To analyze the WGD events among DXWR, *O. brachyantha*, *Z. mays*, *O. sativa*, *Triticum aestivum* and *Arabidopsis thaliana*, their protein sequences were aligned against themselves with Blastp (E-value ≤ 1e−10) to acquire conserved paralogs in each species. Then, the respective collinear blocks of these species were identified with MCScanX. Finally, potential WGD events in each genome were evaluated based on their 4DTv (Kimura, 1980) and Ks distribution (Blanc and Wolfe, 2004).

### Evolution analyses

The nucleotide and amino acid sequences of nine species (*O. brachyantha*, *O. rufipogon*, *Z. mays*, *S. italica*, *O. sativa*, *T. aestivum*, *Brachypodium distachyon*, *Oryza meyeriana var. Granulata* and *A. thaliana*) were downloaded. All-to-all BLASTP with an E-value threshold of 1e-5 was applied to determine the similarities between protein sequences for all the species, and genes were classified into orthologues, paralogues and single copy orthologues (only one gene in each species) using OrthoMCL (v2.0.9) (Li et al., 2003). For the genes of unique family, the GO and KEGG enrichment analysis were performed to reveal the function of these unique genes.

Molecular phylogenetic analysis was conducted using all the single copy orthologues genes, and each gene family for multiple sequence alignment used Mafft (Katoh and Standley, 2013) and curated the alignments with Gblocks v0.91b (Castresana, 2000). The phylogenetic tree was built based on the PROTGAMMAAUTO model and a bootstrap of 1,000 by RAxML (v 8.2.11) (Stamatakis, 2006). A. thaliana was set as the outgroup. MCMCTREE in PAML v4.9e was used to estimate the divergence times (Yang, 2007). Two fossil calibration times were obtained from the TimeTree database (http://www.timetree.org/), including divergence times of 147.97–172.96 Mya and 9.89–21.38 Mya.

### Comparative transcriptome analysis

Transcriptome datasets under normal and cold treatment were downloaded from NCBI (accessions:SRP026336) (Shen et al., 2014). NGSQC Toolkit (Patel and Jain, 2012) was used to remove the adapter sequences and low quality sequence reads. Clean reads were aligned with the reference genome DXWR by Hisat2 v2.0.5 (Kim et al., 2015). FeatureCount (Liao et al., 2014) was used to calculate the read count. Differentially expressed genes (DEGs) was identified with the thresholds |log2(FoldChange)| > 1 and padj <0.005 by edgeR.

### SV calling and collinear analysis

To investigate the structure variations among the wide and cultivated rice, minimap2 (Li, 2021) was selected for alignment and Smartie-sv pipeline (https://github.com/zeeev/smartiesv) was used to call SVs (Kronenberg et al., 2018). Collinear analysis among different genomes was analyzed using MCScanX (Wang et al., 2012). For verification with single chromosome, MUMmer (Kurtz et al., 2004) was used to display the detail information.

## Results

### Genome sequencing and assembly

**FIGURE 1:**
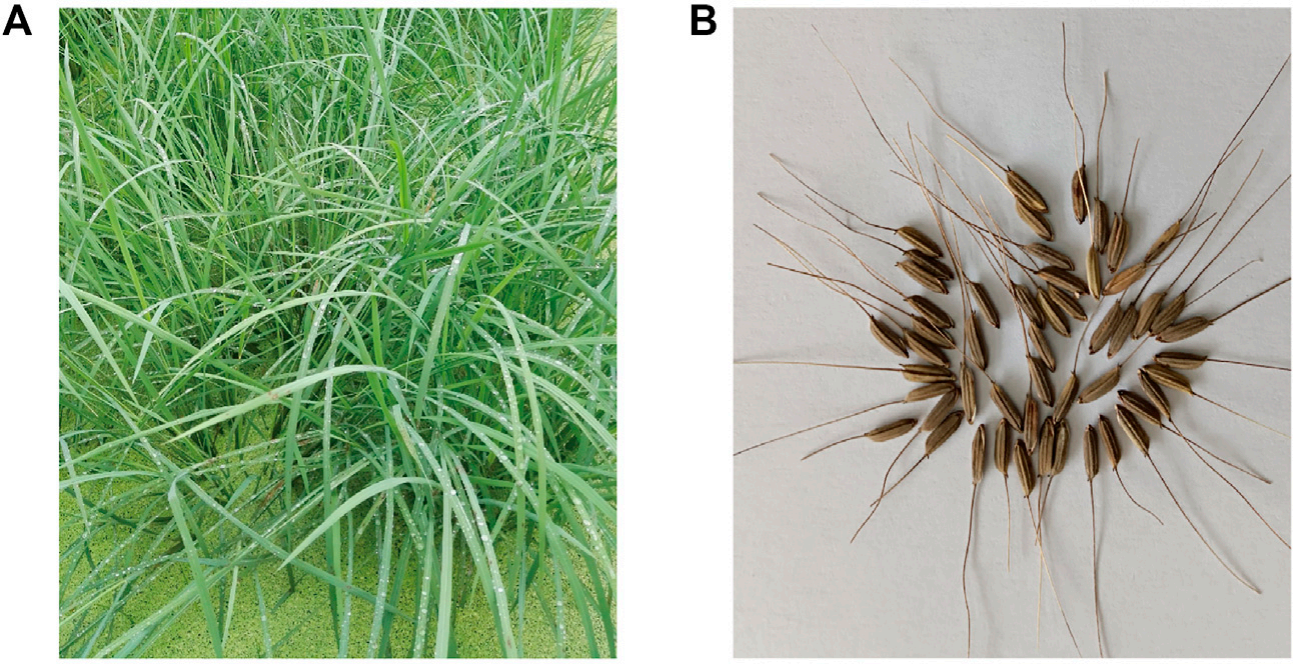
The plants **(A)** and seeds **(B)** of DXWR.

To obtain a high-quality genome assembly for DXWR (Figure 1), three methods including Illumina short read sequencing, Nanopore long read sequencing and Hi-C chromosome conformation capture were used. After filtering, a total of 18.65 Gb clean PE reads (∼45x) were yielded for genome survey and correction, 58.41 Gb Nanopore long reads (∼140x) with reads N50 of 33.36 kb were obtained for genome assembly, and 44.58 Gb clean Hi-C reads (∼108x) were used for chromosome construction (Supplementary Table S1). The genome size was surveyed with FindGSE and GenomeScope based on Illumina PE reads, and the predicted size ranged from 374.82 to 421.16 Mb. The heterozygous and repeat rate were 0.5% and 35%, respectively (Supplementary Figure S1; Supplementary Table S2).

For genome assembly, the corrected nanopore reads were assembled into 233 contigs with 414.59Mb, and the contig N50 size was 5.17 Mb. Further, the polished genome sequences were aligned to the NT database, and two contigs with a total of 1,126,901 bp were filtered out as mitochondrion and chloroplast genomes. Therefore, the final assembly for nuclear genome was 413.46 Mb with 231 contigs and the contig N50 was 5.18 Mb (Table 1), which was very close to the predicted genome size.

**TABLE 1:** Genome assembly statistics and post-processing of Dongxiang wild rice.

Genome assembly	Total assembly size of contigs/scaffolds (bp)	Number of contigs/scaffolds	N50 contig/scaffold length (bp)
Smartdenovo	405,743,318	233	5,062,292
Smartdenovo + Racon 3× + Nextpolish 4×	414,589,793	233	5,177,887
Nuclear genome	413,462,892	231	5,177,887
Nuclear genome + Hi-C	413,480,492	66	33,492,066

**FIGURE 2:**
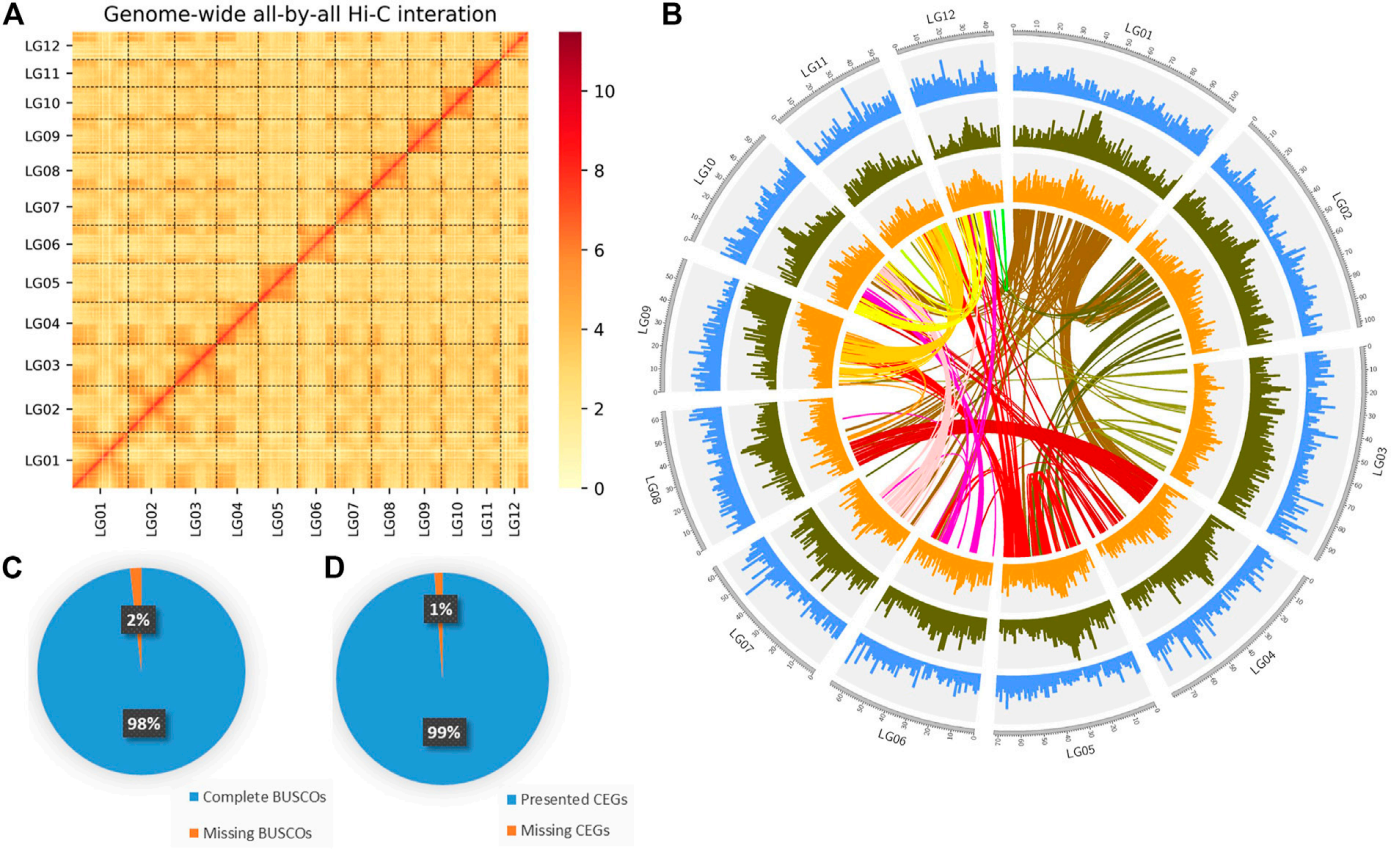
Genome assembly of DXWR. **(A)** The genome contig contact matrix. The blocks indicated the contacts between linkage groups, color depth indicated the degree of contacts. **(B)** Circos plot of genomic features. The tracks from the outermost to innermost are: chromosome, gene density, repeat density, GC content, collinear genes of DXWR. **(C,D)** Verification of genome integrality based on the BUSCO and CEGMA.

After mapping, 62,511,242 valid paired reads were used for Hi-C scaffolding analysis, and the assembled 231 contigs were clustered into 12 groups, which were further ordered and oriented into chromosomes. And 97.39% (402,670,344 bp) of the total contig bases (413,462,892 bp) were reliably anchored to the 12 chromosomes (Figure 2A). Finally, the nuclear genome size was 413,480,492 bp with a scaffold N50 of 33.49 Mb (Figure 2B; Table 1).

To evaluate the completeness genome of the nuclear genome, BUSCO v3.0.1 was performed by using embryophyta_odb10 database with default parameters to search single-copy orthologs genes. Approximately 98.25% of the orthologs genes were found in the assembly (Figure 2C; Supplementary Table S3). Meanwhile, Core Eukaryotic Genes Mapping Approach (CEGMA) was also used, and a total of 245 core genes were identified, which was 98.79% of the eukaryotic core genes (the complete set was 248) (Figure 2D). These results showed that the genome assembly of DXWR was highly complete and robust. Moreover, the accuracy was checked by the SNPs and Indels which were detected from alignment with the nuclear genome. Finally, A total of 3,269 homozygous SNPs (0.000233% of the assembled genome) and 964 homozygous Indels (0.000343% of the assembled genome) were identified with more than ×10 sequencing depth. The accuracy of the assembled genome was up to 99.999%, which suggested the high accuracy of the assembly.

### Genome annotation

**FIGURE 3:**
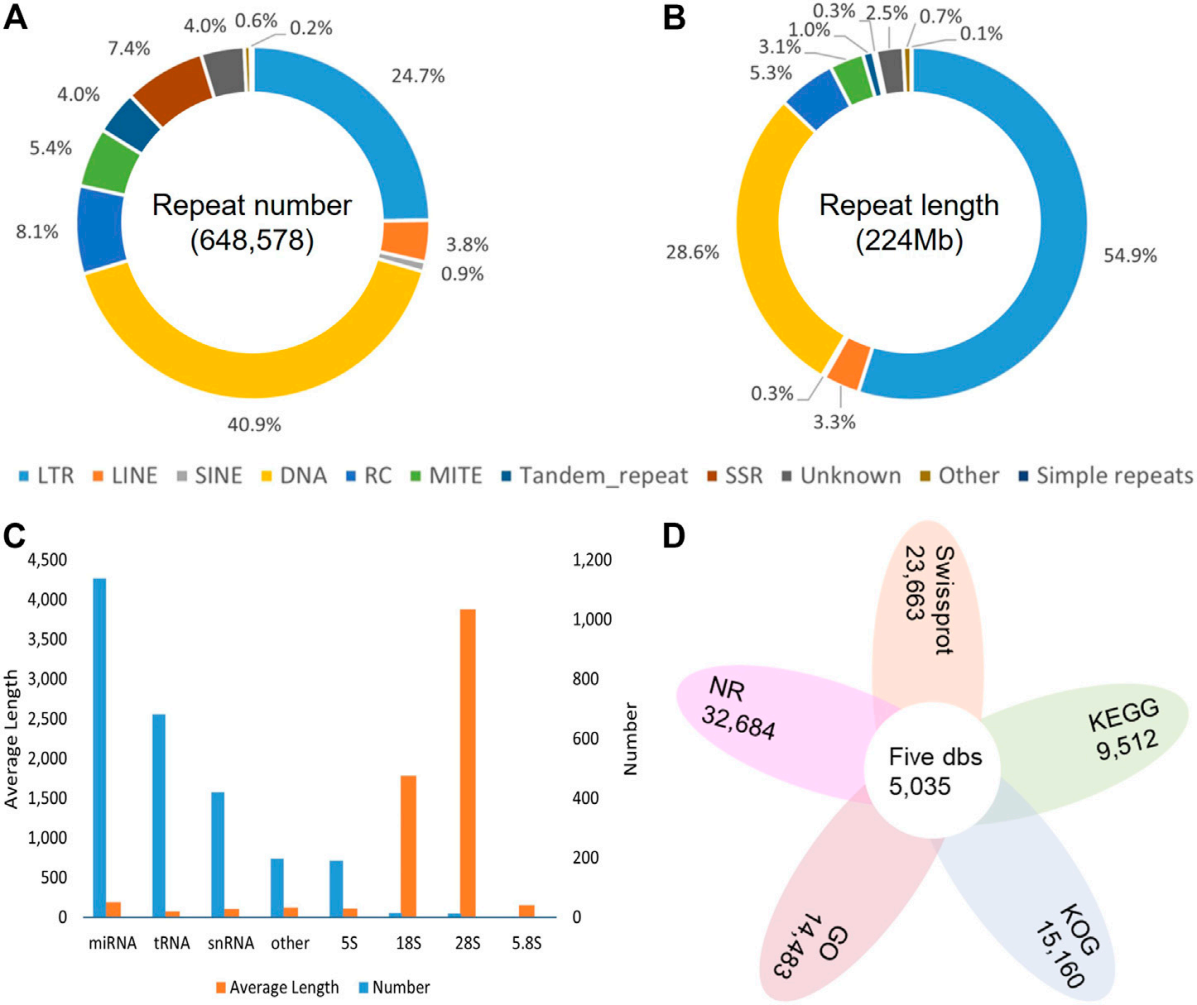
Genome annotation of DXWR. **(A)** The proportion of repeat classes in the total number of annotated repeats. **(B)** The proportion of repeat classes in the total length of annotated repeats. **(C)** The Number and average length of Non-coding RNAs. **(D)** The gene numbers annotated by public databases.

Overall, 54.10% in the DXWR genome (223,689,067 bp) were identified as repetitive sequences. Among all the repeat elements, transposable elements (TEs) were the main types, accounting for 51.67% (213,636,330 bp). In terms of TEs, the dominant type was DNA transposon, but the longest total length was long terminal repeat (LTR), with the ratio up to 29.71% (122,836,029 bp) of the genome (Figures 3A,B; Supplementary Table S4). For non-coding RNAs, a total of 222 rRNA, 1,839 small RNA, and 683 tRNA were identified (Figure 3C; Supplementary Table S5). For protein-coding genes, the results of three methods were merged, and finally 35,942 protein-coding genes were predicted, with an average CDS length of 1,128.28 bp and an average exons number of 4.63 for each gene. To check the completeness of the genome annotation, BUSCO v3.0.1 was also used with default parameters. The results showed that 96.80% of the orthologs genes were found in DXWR annotated gene set (Supplementary Table S3), indicating most conserved genes of DXWR were predicted completely. To better understand the function of predicted genes, a variety of databases were used. In total, 33,862 (94.21%) genes were successfully assigned to at least one public functional database. Specifically, 90.94%, 65.84%, 42.18%, 40.3%, 26.46% of the total genes were mapped into the NR, Swissprot, KOG, GO and KEGG databases, respectively (Figure 3D; Supplementary Table S6). And 5,035 (14.01%) genes were annotated simultaneously by these five databases.

### Comparative genomics and evolutionary analysis

**FIGURE 4:**
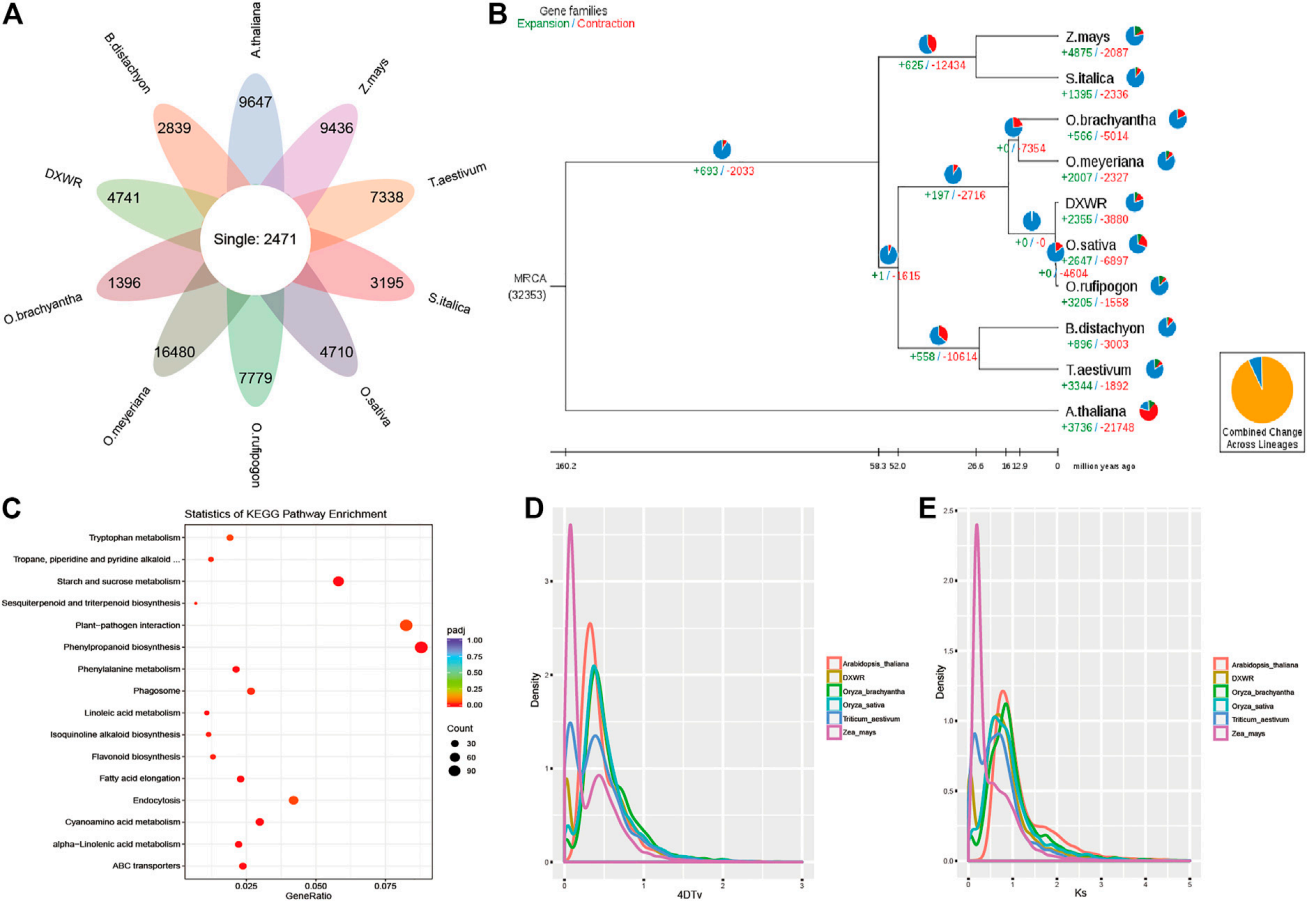
Comparative genomics and evolutionary analysis. **(A)** The orthologues genes and specific genes of ten species. **(B)** Phylogenetic relationship and expanded/contracted gene families. **(C)** KEGG enrichment results of the expanded gene families. **(D,E)** 4DTv and Ks distribution.

The protein sequences of DXWR and nine species were selected for comparative genomics analysis, and 2,471 single copy orthologues genes were identified among them (Figure 4A). For unique genes, the number ranged from 1,396 to 16,480 in these species, and there were 4,741 unique genes in the genome of DXWR. Then GO and KEGG enrichment analysis was used to predict the function of these unique genes, and the results showed that hydrolase activity, polysaccharide binding and kinase activity were enriched terms in the molecular function category, whereas the terms including defense response and recognition of pollen in the biological process category were highly enriched. Meanwhile, KEGG enrichment analysis showed that plant-pathogen interaction and plant hormone signal transduction pathways were the significantly enriched ones. It suggested that DXWR could resist against various biotic and abiotic stresses, which would be an invaluable gene pool for the genetic improvement of modern rice cultivars.

For evolution analysis, 2,471 single copy orthologues genes were used to construct the phylogenetic tree with *Arabidopsis* as the outgroup. It was obvious that *O. sativa* and W1943 were the most closely relative of DXWR. According to the gene families and phylogenetic tree (Figure 4B), 2,355 expanded gene families and 3,880 contracted gene families were identified in the genome of DXWR. The KEGG enrichment analysis of the expanded gene families revealed that plant-pathogen interaction, phenylpropanoid biosynthesis, starch and sucrose metabolism were the most enriched pathways (Figure 4C). As to contracted gene families, response to oxidative stress, peroxidase activity and aminoacyl-tRNA ligase activity terms were enriched.

Whole Genome Duplication is an important event in the history of biological evolution, which has great significance in the origin of species and the expansion of genomes, therefore, we investigated the WGD event in DXWR by comparing with other five symbolic species. And the results of 4DTv and Ks distribution (Supplementary Table S7) suggested that DXWR experienced two recent WGD events, just like its close relative *O. brachyantha* and *O. sativa* (Figures 4D,E).

### Comparative analysis of differentially expressed genes

To investigate expression changes under cold stress in DXWR, RNA-seq data from normal and cold treatment was compared. In total, 1801 DEGs were determined with the thresholds |log2(FoldChange)| > 1 and padj <0.005, among them, 1,072 DEGs were up-regulated and 729 DEGs were down-regulated. The KEGG enrichment of DEGs showed that they gathered in the process of photosynthesis, carbon metabolism, glyoxylate and dicarboxylate metabolism, biosynthesis of amino acids, pentose phosphate pathway, carotenoid biosynthesis and linoleic acid metabolism. Moreover, 43 DEGs were overlapped with contracted genes, while 340 DEGs were determined in expanded gene families of DXWR (Supplementary Table S8), which would be candidate genes for DXWR to enhance the cold resistance. Actually, there were a number of genes that had been verified as cold-response genes, like potassium transporter 1, sugar transport protein MST6-like, calmodulin-like protein 5, glycerophosphodiester phosphodiesterase GDPDL3-like, polyol transporter 5, CBL-interacting protein kinase 5, protein NRT1/PTR FAMILY 6.2 and so on.

### Structural variation and collinear analysis

**FIGURE 5:**
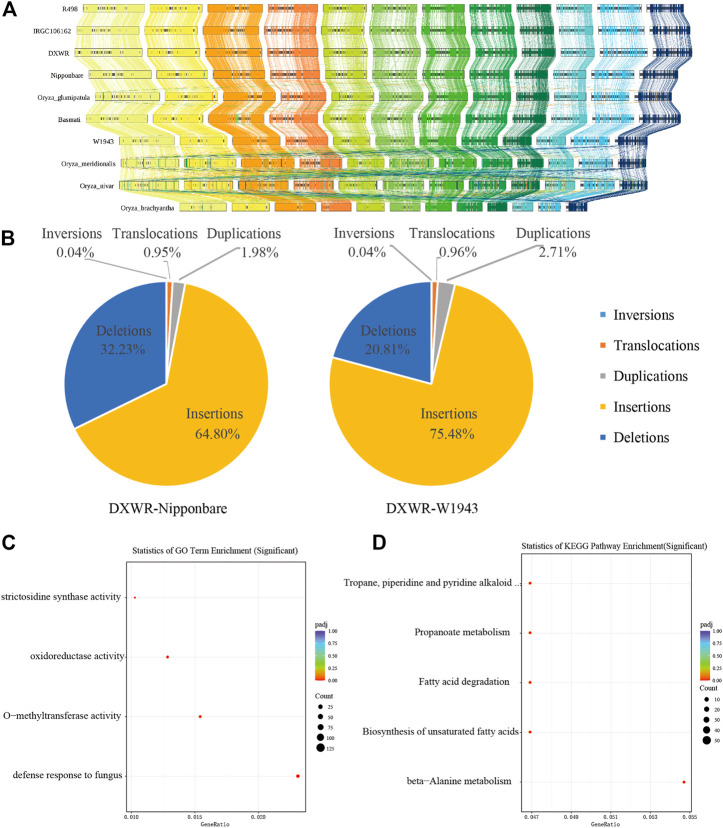
The genomic variations of DXWR. **(A)** Synteny analysis of genes in ten crops of *Oryza* genus. **(B)** The structure variation of DXWR and Nipponbare and W1943. The results of GO enrichment **(C)** and KEGG enrichment **(D)** analysis of genes involved in inversion in DXWR.

SVs are a major source of genetic diversity (Hollister, 2014; Wang et al., 2018; Ho et al., 2020). In order to understand the structural variation of DXWR, we conducted a collinearity analysis of ten species of *Oryza* including DXWR (Figure 5A). The genome sequences of the *Oryza* genus had relatively high collinearity. Compared with the other nine *Oryza* species, DXWR and Nipponbare had a nearly 6 Mb inversion on chromosome 6 (Figure 5A).

Using wild rice DXWR as a reference genome, we aligned cultivated rice Nipponbare (*O. sativa*) and wild rice W1943 (*O. rufipogon*) genomes with DXWR genomes, respectively. As a result, DXWR had more collinear regions and longer collinear lengths with W1943 (Figure 4B). Insertion and deletion accounted for the majority of structural variants, whereas inversion proportion was the least one (Figure 5B). At the same time, we found SNPs and SVs between DXWR and W1943 were less than those of DXWR and Nipponbare, which was consistent with the evolutionary relationship of the three, that is, DXWR and W1943 were more closely related, and more distantly related to cultivated rice (Figure 4B).

Chromosomal inversion is a structural variation that often contributes significantly to evolution, and its appearance is often related to biological processes such as biological adaptive phenotype and differentiation (Arostegui et al., 2019; Wellenreuther et al., 2019). Compared with the cultivated rice Nipponbare, it was discovered that there are 154 inversion regions in DXWR, of which 105 inversion fragments contained 1,061 genes (Supplementary Table S9). These genes were unevenly distributed on 12 chromosomes, among which chromosome 6 and chromosome 11 had the most genes, 284 and 268 respectively. GO enrichment analysis of these 1,061 genes revealed that these genes were associated with denfense response to fungus (Figure 5C), while KEGG enrichment analysis found that these genes were connected to fatty acid metabolism, such as fatty acid degradation and Biosynthesis of unsaturated fatty acids (Figure 5D). This was similar to the enrichment analysis results of DXWR expansion genes, which were also related to plant-pathogen interactions and fatty acid metabolism.

**FIGURE 6:**
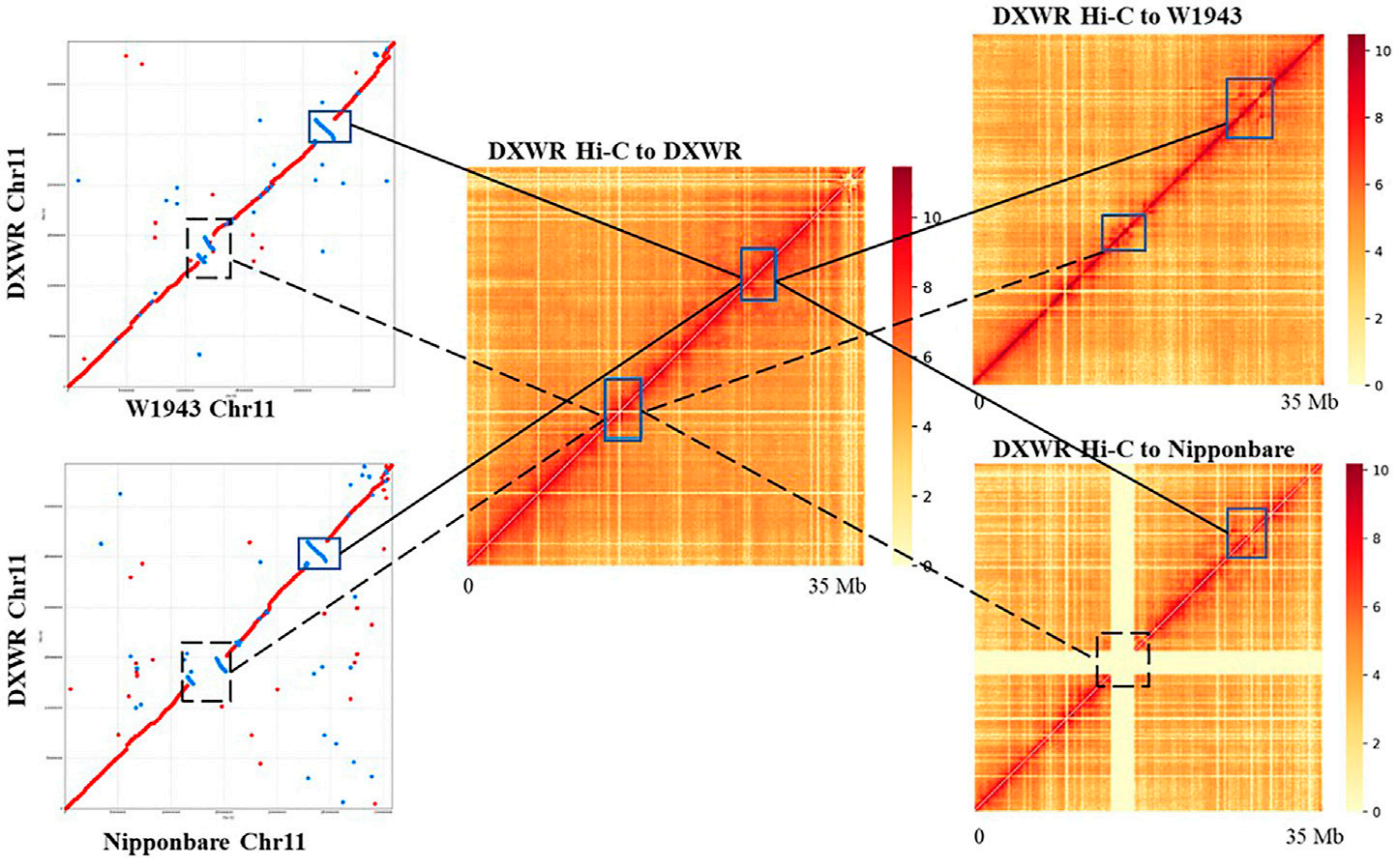
The inversions between DXWR and Nipponbare and W1943 supported by Hi-C contact maps.

There are three inversions larger than 500 kb in chromosome 11 of DXWR (Chr11: 12,364,796–13,088,674, Chr11: 13,518,190–14,923,590, Chr11: 24,597,628–26,486,542). Compared with the other two wild rice (*O. rufipogon*) and other *Oryza* rice, these three inversions existed specifically in DXWR (Figure 5A), which may be one of the reasons for the differentiation of DXWR. We verified the authenticity of these three inversions using Hi-C data. The Hi-C data of DXWR was aligned to the genomes of W1943 and Nipponbare, respectively. The heat map signal can clearly find that the inversion occurred at the corresponding position of chromosome 11 of W1943 and Nipponbare, confirming the authenticity of the inversions of the corresponding position of chromosome 11 (Figure 6). The longest inversion (Chr11: 24,597,628–26,486,542) of the three inversions was 1,888,914 bp in length and involved a total of 176 genes. The enrichment analysis of these genes found that the main functions described defense response to fungus, ADP binding and linoleic acid metabolism. There are six genes (evm.model.Contig 25.47–51 and evm.model.Contig 25.99) related to defense response to fungus in this inversion region, of which the first five are tandem genes, and these five genes are all related to defense response to bacterium. At the same time, unsaturated fatty acids can enhance the cold resistance of plants, so this inversion may be related to the disease and cold resistance of DXWR. Colinearity analysis of ten varieties from *Oryza* genera showed that the SV in Chr11 existed only in DXWR (Supplementary Figure S2). Then we compared this area in detail. We performed collinearity analysis on W1943 (Chr11: 20.88–23.27 Mb), DXWR (Chr11: 24.06–27.26 Mb) and IRGC106162 (Chr11: 24.14–23.26.83 Mb) and confirmed that this SV existed alone in DXWR and absent in W1943 and IRGC106162 (Supplementary Figure S3).

## Discussion

Wild rice is usually regarded as abundant resource for holding genetic diversity, excellent agronomic traits and resistance against stresses (Xu et al., 2011; Zhao et al., 2018). DXWR is a common wild rice located at the northernmost of *O. rufipogon* species. To better understand and exploit the characteristic of DXWR, sequencing the genome is a convenient and effective way, which will provide all the genetic information. In this study, a chromosome-level genome of DXWR was assembled using nanopore long reads and high-through chromatin conformation capture (Hi-C) technology, which could overcome issue of heterozygosity and high repeat rate. After primary assembly, more than 97% of the total contig bases (413,462,892 bp) were correctly anchored into 12 chromosomes. The final nuclear genome was 413.48 Mb with a scaffold N50 of 33.49 Mb, which was a little larger than other published rice genome, and not only DXWR, it was noted that generally the genome size of *O. rufipogon* varieties was larger (Shang et al., 2022), which was caused by higher repeat rates, especially the transposon expansion. Moreover, both BUSCO and CEGMA assessment verified the completeness of the genome, and the high accuracy of the genome was also affirmed by alignment using short reads. For protein-coding genes, a high annotation rate was observed, up to 94.21% genes could be annotated by public functional databases. The high-quality genome and annotation could supply valuable resources for comparative genomics, evolution analysis and genetic breeding of rice.

Based on the high-quality genome assembly, DXWR was compared with other species to understand its character. According to the enrichment analysis of unique genes and expanded gene families, it was noted that plant-pathogen interaction, phenylpropanoid biosynthesis, starch and sucrose metabolism, and plant hormone signal transduction pathways were significantly enriched. The plant-pathogen interaction and defense response would help DXWR survive under biotic stress (Kushalappa and Gunnaiah, 2013). Phenylpropanoid which was a rich source of secondary metabolites in plants, together with plant hormone, played important roles in plant growth, development, and defense (Desmedt et al., 2021; Dong and Lin, 2021). Moreover, starch and sucrose metabolism was the vital pathway in the process of rice grain filling, which could finally affect the yield (Fan et al., 2019; Jiang et al., 2021; Mathan et al., 2021). All these information indicated that DXWR probably had superior disease-resistance and starch synthesis ability.

Like other stress-responsive traits, the cold resistance characteristics of plants are affected by multiple factors and are also controlled by genetics (Xu and Cai, 2014; Fang et al., 2017). As we know, cold resistance is related to the content of unsaturated fatty acids in plants. The increase of unsaturated fatty acids in membrane lipids will reduce the temperature of phase transition of membrane lipids, so the cold resistance of plants can be improved by increasing the degree of unsaturation of fatty acids (Khodakovskaya et al., 2006; Matteucci et al., 2011; Soria-Garci et al., 2019). Overexpression of the chloroplast omega-3 fatty acid desaturase gene (LeFAD7) in tomato (Lycopersicon esculentum Mill.) can increase the content of unsaturated fatty acids and enhance the resistance to low temperature stress (Liu et al., 2008). Overexpression chloroplast ω- 3 fatty acid desaturase gene can also enhance the cold tolerance of transgenic tobacco (Khodakovskaya et al., 2006). The increase of small molecular substances and soluble substances is one of the cold resistance responses of plants, and plants with strong cold resistance will accumulate more soluble sugars (Wei et al., 2017; Zhao et al., 2019; Liu et al., 2021). These soluble sugars have a certain protective effect on preventing protein denaturation after dehydration, and intercellular sugars will alleviate low temperature damage by affecting the growth of ice crystals. Liang et al. (2021) reported that the increasing of soluble sugar in grapes can enhance the cold resistance of grapes. DXWR is the wild rice with the most northward distribution and the strongest cold resistance discovered so far. It can resist low temperature and cold wave for a long time in the seedling and heading stage. Analysis of its genome and comparative transcriptome under normal and cold treatment found that genes related to phenylpropanoid biosynthesis, linoleic acid metabolism and starch and sucrose metabolism were significantly expanded and differentially expressed in DXWR, which may promote the cold resistance of DXWR. The discovery of cold resistance-related genes in wild rice is of great significance for understanding the cold resistance mechanism of wild rice and cultivating strong cold resistance rice varieties.

SNPs have long been considered to be a significant component of genetic variation, but now there is increasing evidence that structural variation is also an important part of genetic variation (Alonge et al., 2020; Liu et al., 2020b; Qin et al., 2021). Structural variation may have the addition or deletion of DNA information, such as insertion, deletion, or duplication, but it may not lead to the increase or deletion of DNA information, such as inversion, transposition in an individual (Fu et al., 2016; Yang et al., 2019b). SVs are a key and pervasive force driving genetic diversity and contribute to important agronomic traits in crops (Yang et al., 2019a; Yang et al., 2019b). In tomato, SV has been shown to relate with fruit size and flavor, disease response, and the plant’s ability to detect pathogens (Jobson and Roberts, 2022). Copy number variants (CNVs), one important type of SV, has been shown to play an important role in the adaptive response of plants, by regulating development, and by increasing resistance to biotic and abiotic stresses (Francia et al., 2015). SVs in maize, sorghum and rice have been shown to be associated with plant disease resistance, and stress responsive genes in soybeans have also found related with SVs (Saxena et al., 2014). Published studies have shown that SVs play an important role in the domestication of rice from wild rice to cultivated rice, and peaks of SV divergence were concentrated in genes associated with domestication (Kou et al., 2020). The appearance of inversion is often related to evolution and biological processes, such as biological adaptive phenotypes and differentiation. In the chromosome 11 of DXWR, there was an inversion region close to 2 Mb, which is consistent with the results obtained by Mao et al. (2015) using SLAF marker. Compared with the other nine *Oryza* genus plants, this inversion was specific to DXWR and this region was related to 176 genes. The enrichment analysis showed that these genes were closely related to defense response to fungus and linoleic acid metabolism, which may be the reason for the differentiation and characteristics of DXWR.

## Conclusion

In this research, a high-quality chromosome-level genome of wild rice DXWR was obtained using Nanopore sequencing and Hi-C technology, which could provide elaborate genomic information for evolution and genetic breeding. Here the comparative genomics and transcriptomics analysis had indicated that DXWR probably had superior disease and cold resistance. Moreover, when compared to other cultivated rice, DXWR exhibited certain distinct inversions. These inversions were also connected to defense response to biotic stress and may be responsible for the characteristics of DXWR. Overall, the genome assembly of DXWR would help us to better understand and utilize the characteristic of this wild rice, which is critical for future genetic breeding in this crop.

## Data Availability

This Whole Genome Shotgun project has been deposited at GenBank under the accession PRJNA641241. All sequencing data are available at NCBI Sequence Read Archive SRR12104676, SRR12102354 and SRR12102351. The genome assembly and annotation files are available at figshare https://doi.org/10.6084/m9.figshare.20072117.v1.

## Supplementary material

SUPPLEMENTARY FIGURE S1 K-mer and coverage distribution of DXWR.

SUPPLEMENTARY FIGURE S2 The collinearity relationship between ten rice varieties in the Oryza genus.

SUPPLEMENTARY FIGURE S3 The collinearity relationship between W1943 (Chr11: 20.88–23.27 Mb), DXWR (Chr11: 24.06–27.26 Mb) and IRGC106162 (Chr11: 24.14–23.26.83 Mb).
