# Supplementary figures and images for: Chromosome-level genome assembly of Dongxiang wild rice (Oryza rufipogon) provides insights into resistance to disease and freezing
Source: Front Genet. 2022 Nov 15;13:1029879. doi: 10.3389/fgene.2022.1029879 (PMC9707695; doi:10.3389/fgene.2022.1029879)

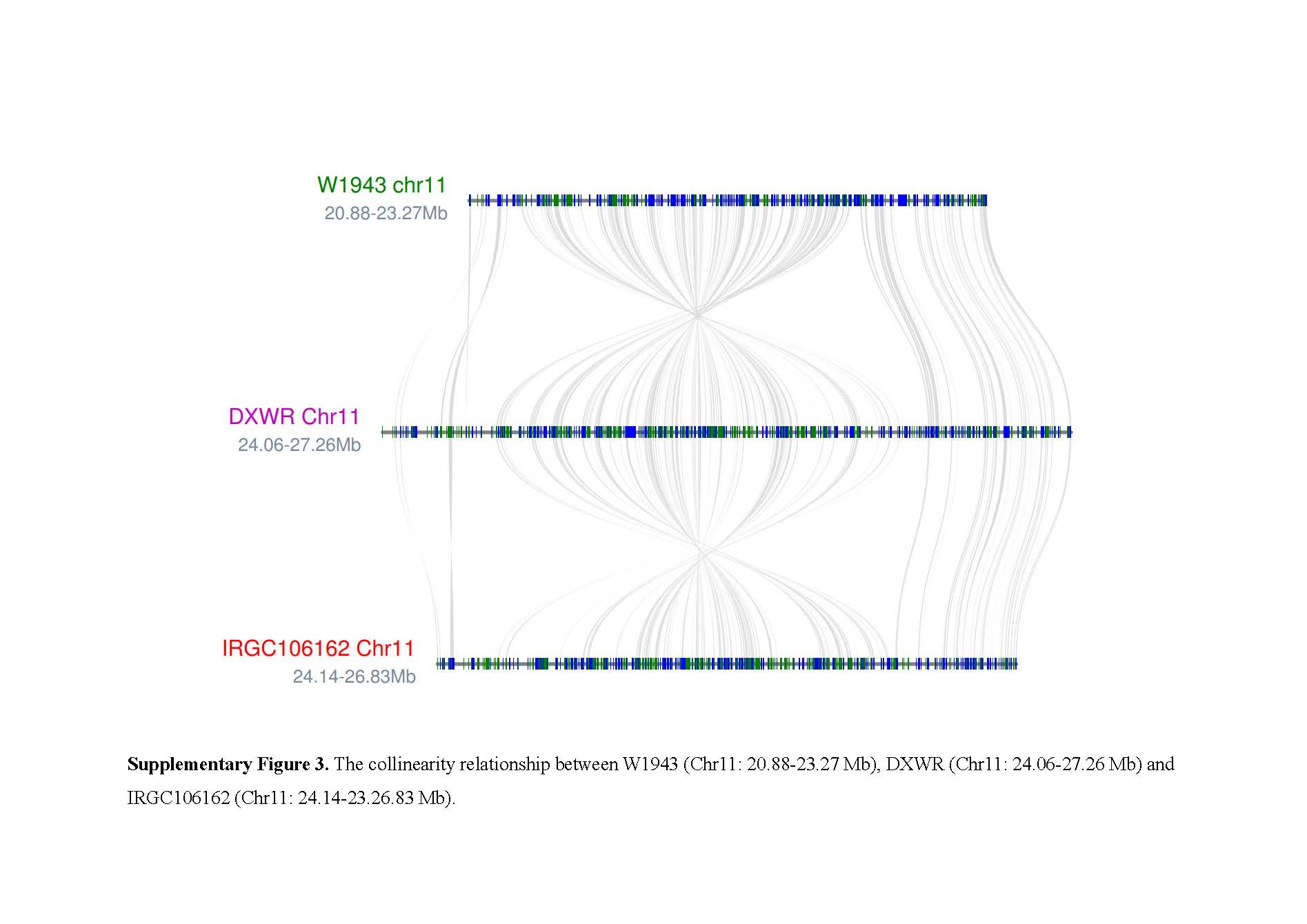

Supplement: Supplementary file 1 [file Image3.JPEG]

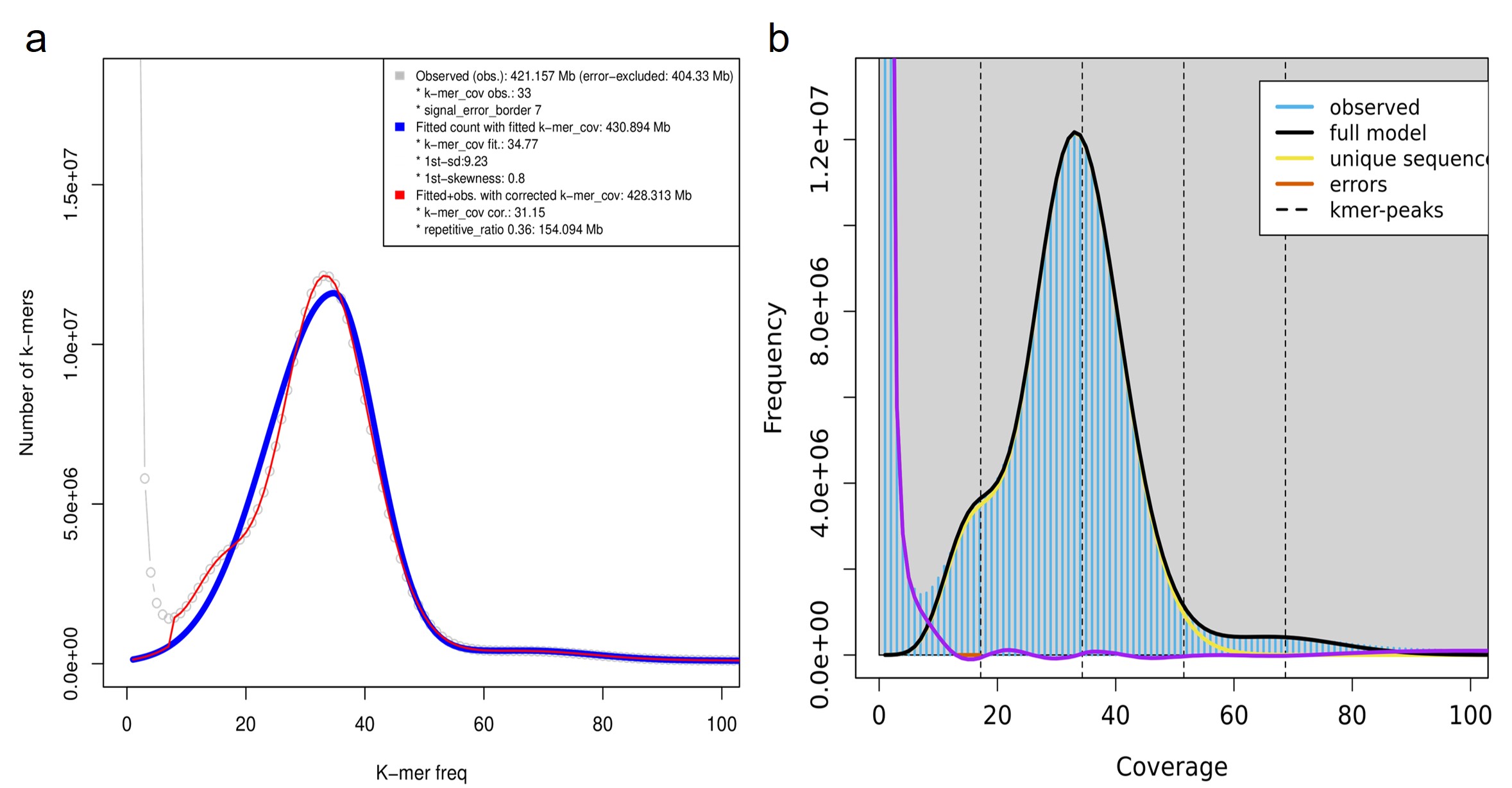

Supplement: Supplementary file 2 [file Image1.JPEG]

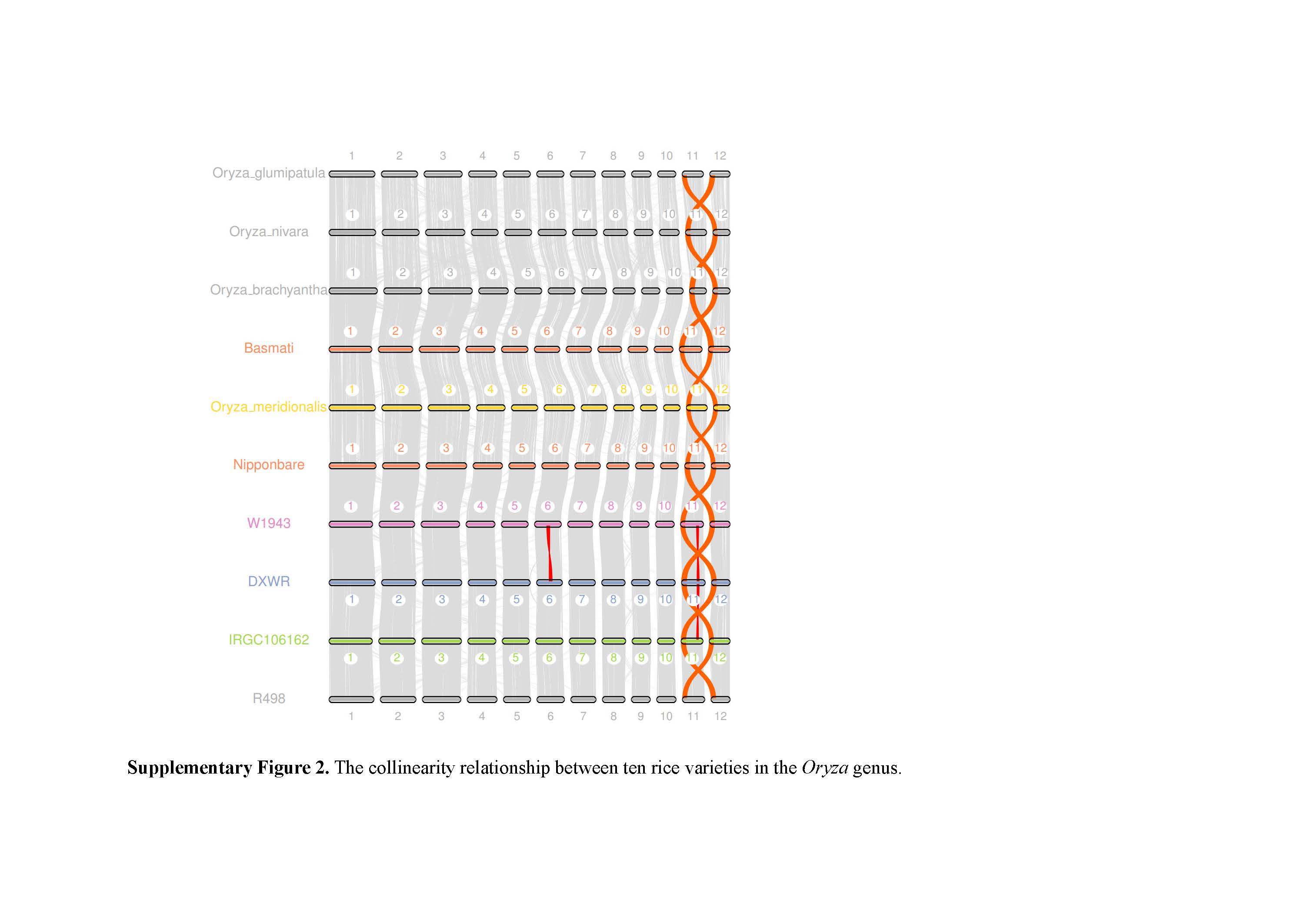

Supplement: Supplementary file 3 [file Image2.JPEG]
